# Rare features associated with Mobius syndrome: Report of two cases

**DOI:** 10.15171/joddd.2017.012

**Published:** 2017-03-15

**Authors:** Rumela Ghosh, Vikram Shetty, Shruthi Hegde, G Subhas Babu, Vidya Ajila, Nanda Kishore P, Mithula Nair

**Affiliations:** ^1^Department of Oral Medicine and Radiology, A B Shetty Memorial Institute of Dental Sciences, Nitte University, Mangalore, India; ^2^Nitte Meenakshi Institute of Craniofacial Surgery, K.S.Hegde Medical College and Hospital, Nitte University, Mangalore, India; ^3^Department of Orthodontics, A B Shetty Memorial Institute of Dental Sciences, Nitte University, Mangalore, India

**Keywords:** Congenital, facial nerve, mobius syndrome, palsy, strabismus

## Abstract

Mobius syndrome is a rare congenital disorder with the preliminary diagnostic criteria of congenital facial and abducent nerve palsy. Involvement of other cranial nerves, too, is common. Prevalence rate of this syndrome is approximately 1 in 100,000 neonates. It is of unknown etiology with sporadic occurrence. However, data regarding the occurrence rate in India is limited. Features such as orofacial malformations, limb defects, and musculoskeletal, behavioral, and cognitive abnormalities might be associated. A thorough evaluation to identify the condition and establishing an adequate treatment plan is of utmost important in this condition. We are reporting clinical and radiographic features of Mobius syndrome in two cases along with unusual findings of limb and neck deformity.

## Introduction

Mobius syndrome is a congenital facial weakness combined with abnormal ocular abduction in most studies. In 1888, a German neurologist, Paul Julius Mobius, first described the clinical entity of Mobius syndromes.^1^ He reported patients with congenital and delayed facial and abducent nerve palsy. However, von Graaefe was the first to report patients with facial diplegias.^1^ Hallmarks of Mobius syndrome mainly consist of paresis of 6th and 7th nerves. It may or may not be associated with paralysis of other cranial nerves (III, V, VI, IX, X) and abnormalities of the limb (syndactyly, brachydactyly or absence of digits), chest wall, spine and soft tissues.

This condition is considered to be one of the infrequent syndromes associated with disorders of oromandibular limb hypogenesis. Prevalence rate of this syndrome is approximately 1 in 100,000 neonates.^2^ The present report describes the features of two cases with this rare syndrome.

## Case report

### Case 1

A 20-year-old female patient reported to the Department of Oral Medicine and Radiology with a chief complaint of inability to close her mouth since birth. Her mother stated that she was also unable to close both eyes since birth and there was difficulty in suckling. Her milestones were normal and there was no history of prenatal or postnatal trauma. Her medical history revealed that she had visited a medical hospital previously for augmentation of the upper lip at the age of 15 years and breast augmentation at the age of 18 years. Intelligence quotient (IQ) evaluation revealed normal IQ level of the patient. There was no history of consanguineous marriage between the parents.

**Figure 1.**
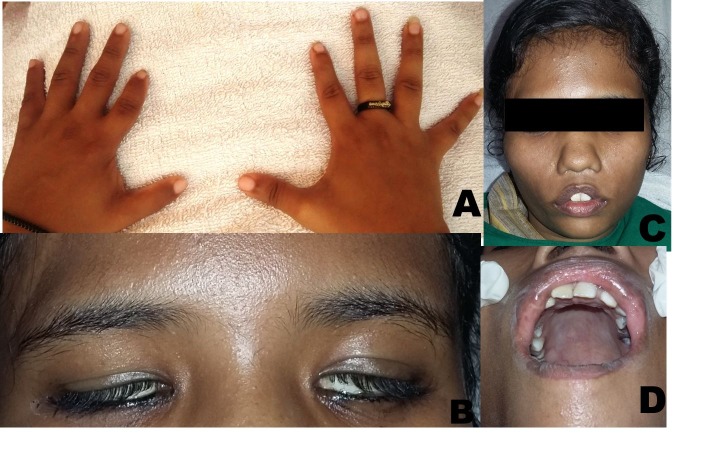
BImage shows shortened index finger on both hands (A); clinical picture shows inability to close eyes (B); the image shows incompetent lips, scar mark over the upper lip (C); intraoral picture showing a high-vault palate (D).

On general examination, all the vital signs were within normal limits. No abnormality was detected in lower limbs whereas shortening of the index finger was noted in both the upper limbs (Figure 1 A). Her sensory functions were normal. She was unable to close both eyes (Figure 1 B) and raise the eyebrows. There was absence of wrinkles on her forehead (Figure 1 C). The patient was neither able to whistle or blow air between her cheeks nor was able to smile. She was not able to completely abduct her eyes and was unable to rotate her eyeballs upwards or downwards and could only partially move it in medial and lateral directions. Other ocular manifestations were the presence of ptosis of the right eye and strabismus. Other facial and intraoral features were wide nasal apertures, incompetent lips (Figure 1, C), scar mark over the upper lip and a high-vault palate (Figure 1, D).

**Figure 2.**
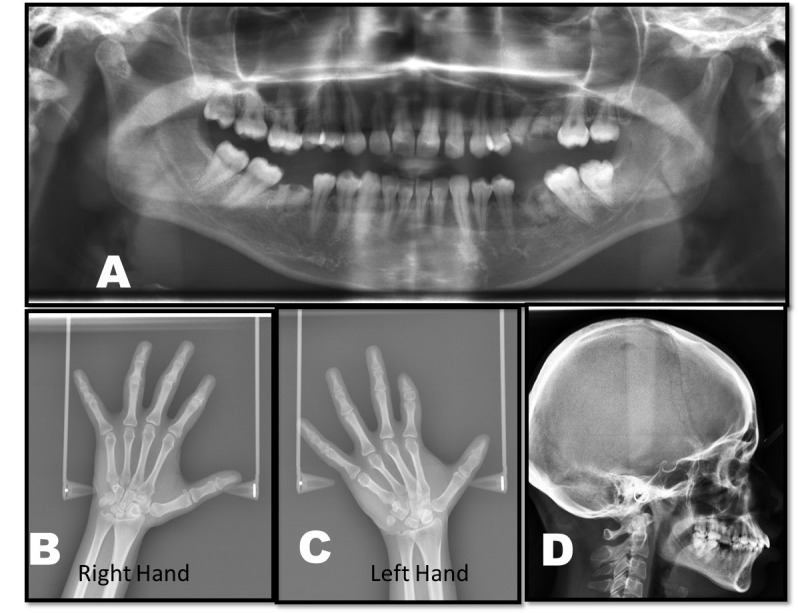
Panoramic view showing decayed tooth with respect to maxillary left second premolar, first molar and mandibular right and left first molar (A). Hand wrist radiograph showing short middle phalanges in the index finger of both hands (B and C). Lateral cephalogram shows no abnormality (D).

Based on history given by the patient and features shown on clinical examination, the case was diagnosed as Mobius syndrome. Routine hematological investigations were within normal limits. Panoramic view showed decays with respect to maxillary left second premolar, first molar and mandibular right and left first molar (Figure 2, A). Wrist radiographs showed short middle phalanges in the index finger of both hands (Figure 2, B and 2, C). A lateral cephalogram showed no abnormality (Figure 2, D). Correction of incompetent lips was planned using a multidisciplinary approach.

### Case 2

**Figure 3.**
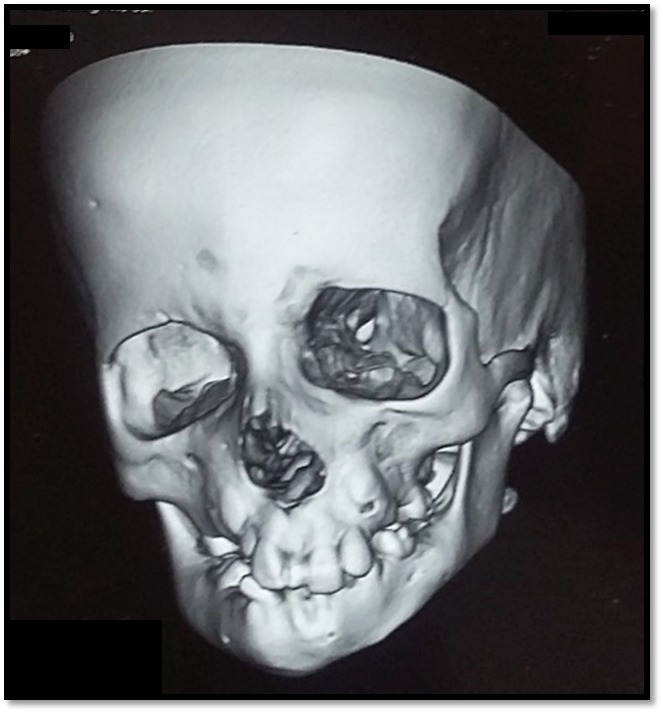
3D CT reconstruction showing orbital dystopia.

**Figure 4.**
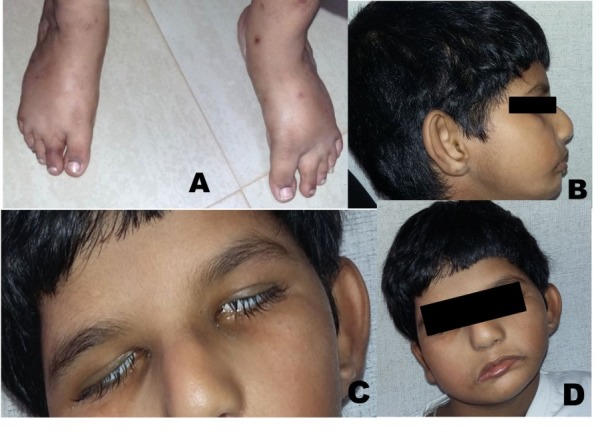
Clinical photograph showing club foot (A). Image shows convex profile, hypoplastic mandible (B). Shows inability to close eyes (C). Clinical photograph shows low set ears, impaired facial expressions and absence of forehead wrinkling and torticollis (D).

A 10-year-old male reported to the Department of Oral Medicine and Radiology with a chief complaint of inability to close his mouth since birth. His parents stated that he was also unable to completely close his eyes since birth. His medical history revealed that he was diagnosed with orbital dystopia. Computed tomography revealed orbital dystopia (Figure 3). The patient had undergone cranioplasty two years back. Conservative treatment of foot for congenital talipes equinovarus at the age of 4 years was reported. His developmental milestones were delayed. IQ evaluation carried out 2 years back revealed average intelligence level of the patient. Family history was noncontributory. On general examination, features of club foot were observed (Figure 4, A). His vital signs were within normal limits. On extraoral examination, the patient had convex profile, with a hypoplastic mandible (Figure 4, B). He was unable to close eyes (Figure 4, C). He had low-set ears, impaired facial expressions and absence of forehead wrinkling was observed (Figure 4, D). Torticollis was also noted (Figure 4, D). Intraorally, a high-vault palate, decayed maxillary right and left first permanent molar, and maxillary left first deciduous molar were noted. Based on history and clinical evaluation, a diagnosis of Mobius syndrome was established.

**Figure 5.**
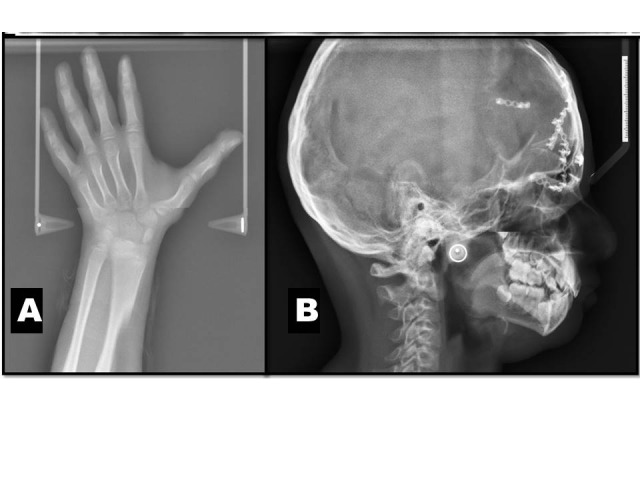
Hand wrist radiograph shows no abnormality (A). Lateral cephalogram shows beaten metal appearance of skull, multiple surgical plates and screws (B).

Wrist radiographs showed no abnormality (Figure 5, A). A lateral cephalogram showed beaten metal appearance of skull and multiple surgical plates and screws (Figure 5, B). A multidisciplinary approach was established for the treatment of the patient. He is under regular follow-up.

## Discussion

Mobius syndrome is described as a syndrome with a combination of congenital palsies of the abducens and facial nerves. Frequently, additional features such as orofacial malformations, limb defects, and musculoskeletal, behavioral, and cognitive abnormalities might be present. These abnormalities result from a rare, complex developmental aberration of the hind brain.^3^It is usually detected during infancy. Both sexes are equally affected.

Abramson et al^4^ classified and graded this syndrome through a retrospective analysis based on the evaluation of the clinical findings of cranial nerve palsy and deformities of the limbs. He used the acronym CLUFT (cranial nerve, lower limb, upper limb, face, and thorax), i.e. cranial nerve discrepancy of either partial or complete 6th or 7th nerve palsies or both; lower extremity findings of clubfoot, ankylosis, longitudinal, or transverse deficits; upper extremity involvement with hypoplasia of digits or failure of formation; structural facial findings of cleft palate, micrognathia, or microtia and thoracic findings of scoliosis, pectoral hypoplasia, or other chest wall deformities.^4^The etiology of the syndrome still remains unknown, and most cases reported are sporadic. However, four genetic loci for Mobius syndrome have been described: MBS1 on chromosome 13q12.2-q13, MBS2 on chromosome 3q21-q22, MBS3 on chromosome 10q21 and MBS4 on chromosome 1p22.^5^ Various other etiologic factors have been associated with it, including vascular interruption in the subclavian artery territory, infections, hyperthermia, trauma and teratogens such as benzodiazepines, thalidomide, alcohol, cocaine, misoprostol and ergotamine.

Mobius syndrome presents a broad spectrum of symptoms and signs in the cranio-maxillofacial region. Major criteria for the diagnosis include non-progressive, congenital, unilateral or bilateral palsy of the 6th and 7th cranial nerves.^6^ Common features are incomplete closure of the eyelid during sleep, inability to smile, drooling of saliva from the mouth, difficulty in sucking, lack of facial movement when crying (masklike facies), asymmetry of the angles of the mouth and indistinct speech secondary to the inability to close the lips, leading to labial sounds.^4^Our cases presented with incomplete closure of the eyelid, inability to smile, difficulty in sucking, masklike facies; in addition, indistinct speech was present in Case 2. Along with these features, oculofacial abnormalities such as strabismus, ptosis, nystagmus, epicanthal folds and tongue abnormalities might be present. Orbital dystopia was noted in Case 2. Limb malformations associated with this syndrome are hypoplasia to aplasia of digits, brachydactyly, syndactyly, ectrodactyly.^5^We noticed shortened index finger in both hands of Case 1. Torticollis and congenital talipes equinovarus was seen in Case 2. In some rare cases mental retardation and autistic behavior of the patient are seen.^7^

There are no specific diagnostic laboratory studies related to the syndrome.^8^We diagnosed Mobius syndrome based on the history and clinical features. A differential diagnosis of Melkersson-Rosenthal syndrome, congenital facial muscular atrophy, muscular dystrophy, cerebral palsy and congenital myopathies were considered.^9^ Our cases were differentiated from Melkersson-Rosenthal syndrome on the basis of absence of lingua plicata and orofacial edema.^10^ In congenital facial muscular atrophy, positive family history and prenatal or postnatal trauma are generally present, which were not reported in our cases. Muscular dystrophy, cerebral palsy and congenital myopathies are primarily diseases of muscular origin whereas Mobius syndrome has muscular pathologies secondary to nerve involvement.^11^

The number of cases reported with the radiological features is very few. CT scan findings mostly include medial deviation of the eyes, hypoplastic or dysplastic brainstem, hypoplastic cerebellum and bilateral calcifications adjacent to the fourth ventricle floor at the level of the 6th cranial nerve nuclei. MRI studies show features of calcification in the pons within the abducens nuclei, hypoplasia of the brainstem and hypoplasia of the cerebellum. Our second case showed orbital dystopia in the CT scan. Neurophysiological studies can be helpful in locating the site of the lesion.^7,12^

Definitive medical treatment is not available for Mobius syndrome, therefore, medical care is only supportive and symptomatic. Parental education plays a major role in the patient’s life. The restoration of motion secondary to the facial nerve palsy that results in mask-like facies and inability to smile is the prime aim of the treatment plan. Management involves plastic surgery reconstruction with muscle transplantation ideally performed at an early age. Ocular surgeries, orthognathic surgeries and surgical corrections for other associated abnormalities may be indicated. Other intervention programs such as physical therapy for congenital orthopedic problems, occupational therapy to help daily activities or speech therapy for patients affected by severe facial nerve paralysis may prove supportive.^13,14^

In Case 1 there was history of treatment with augmentation of the upper lip and breast augmentation. Further correction of lips was planned. A multidisciplinary approach has been established for the treatment of both the patients for aesthetic and supportive care.

As Mobius syndrome usually presents with variable traits, it is of utmost importance to utilize a thorough evaluation process for each child, which might involve consulting an array of professional disciplines. It is the major responsibility of the professionals involved to identify the complications the patient presents and establish an adequate treatment plan. Limited data are available regarding the radiographic features associated with Mobius syndrome. Our patients also presented with features such as shortened index finger, torticollis and congenital talipes equinovarus. The present report highlights the clinical and radiographic features of this rare syndrome, which adds to the existing literature.

## Authors’ contributions

RG, SH, and VS performed the clinical and radiographic examinations. RG, SH and VA drafted the manuscript. GSB, NK and MN contributed to critical revision of the manuscript. All the authors have read and approved the final manuscript.

## Funding

The authors report no funding for this article.

## Competing interests

The authors declare no competing interests with regards to the authorship and/or publication of this article.

## Ethics approval

The authors declare that the patients consent was obtained for the publication of this paper.

## References

[1] Lin KJ, Wang WN (1997). Moebius syndrome: report of case. ASDC J Dent Child.

[2] Bianchi B, Ferri A, Brevi B, Di Blasio A, Copelli C, Di Blasio C (2013). Orthognathic surgery for the complete rehabilitation of Moebius patients: principles, timing and our experience. J Craniomaxillofac Surg.

[3] Broussard AB, Borazjani JG (2008). The faces of Moebius syndrome: recognition and anticipatory guidance. MCN Am J Matern Child Nurs.

[4] Abramson DL, Cohen Jr MM, Mulliken JB (1998). Mobius Syndrome: Classification and Grading System. Plast. Reconst. Surg.

[5] Mobius PJ (1888). Uber Angeborenen doppelseitige AbducensFacialis-Lahmung. Munch Med Wochenschr.

[6] Picciolini O, Porro M, Cattaneo E, Castelletti S, Masera G, Mosca F, Bedeschi MF (2016). Moebius syndrome: clinical features, diagnosis, management and early intervention. Italian Journal of Pediatrics.

[7] Pedraza S, Gamez J, Rovira A, Zamora A, Grive E, Raguer N (2000). MRI findings in Möbius syndrome: correlation with clinical features. Neurology.

[8] Sujatha D, Khader NFA (2015). Moebius Syndrome: A report of 2 cases with review. Sch J Dent Sci.

[9] Srinivasa Raju M, Suma GN, Prakash R, Goel S (2011). Moebius syndrome: A rare case report. JIOMR.

[10] Carolino F, Fernandes M, Plácido JL (2016). Melkersson-Rosenthal syndrome–delay in the diagnosis of an early-onset oligosymptomatic variant. Porto Biomedical Journal.

[11] Kumar D (1990). Moebius syndrome. J Med Genet.

[12] Verzijl HT, Padberg GW, Zwarts MJ (2005). The spectrum of Möbius syndrome: an electrophysiological study. Brain.

[13] De Serpa Pinto MV, De Magalhaes MH, Nunes FD (2002). Moebius syndrome with oral involvement. Int J Paediat Dentistry.

[14] Terzis JK, Noah EM (2002). Möbius and Möbius-like patients: etiology, diagnosis, and treatment options. Clinc in plastic surgery.

